# Cancer systems epidemiology: Overcoming misconceptions and integrating systems approaches into cancer research

**DOI:** 10.1371/journal.pmed.1004027

**Published:** 2022-06-17

**Authors:** Patricia L. Mabry, Nicolaas P. Pronk, Christopher I. Amos, John S. Witte, Patrick T. Wedlock, Sarah M. Bartsch, Bruce Y. Lee

**Affiliations:** 1 HealthPartners Institute, Bloomington, Minnesota, United States of America; 2 University of Minnesota, School of Public Health, Minneapolis, Minnesota, United States of America; 3 Department of Biomedical Data Science, Geisel School of Medicine, Dartmouth College, Hanover, New Hampshire, United States of America; 4 Baylor College of Medicine, Institute for Clinical and Translational Research, Houston, Texas, United States of America; 5 Department of Epidemiology and Population Health, Stanford University, Stanford, California, United States of America; 6 Center for Advanced Technology and Communication in Health (CATCH), CUNY Graduate School of Public Health and Health Policy, New York City, New York, United States of America; 7 Public Health Informatics, Computational, and Operations Research (PHICOR), CUNY Graduate School of Public Health and Health Policy, New York City, New York, United States of America

## Abstract

Patricia Mabry and coauthors discuss application of systems approaches in cancer research.

**Summary points**

- While traditional epidemiological approaches have helped generate important insights about cancer prevention and treatment, they have important limitations and alone cannot bridge the gaps that continue to exist in cancer research and knowledge.
- One shortcoming is the failure to fully account for and characterize the complexity of various systems (e.g., biological, behavioral, social, environmental, and economic) that can lead to cancer and are affected by cancer.
- Systems approaches can help researchers, clinicians, and other decision makers better understand complex systems and address these systems at many levels, ranging from the cellular to the societal scale.
- Systems mapping can shed light on otherwise hidden mental models, and dynamic modeling can enable virtual experimentation—the systematic exploration of counterfactual scenarios not observable in the real world.
- We present and discuss 14 common misconceptions that will need to be overcome in order for systems epidemiology to realize its potential role in cancer prevention and control.
- Examples of systems approaches applied to cancer-related research topics are given to illustrate the utility of systems approaches to transform cancer epidemiology to cancer systems epidemiology.

## Background and significance

Many traditional epidemiological methods are regression-based and attempt to find associations between certain risk factors (e.g., smoking, diet, physical activity) and disease outcomes (e.g., cancer). While these methods help identify factors to explore further, they are not equipped to uncover the complex systems and processes that underlie cancer. That is, they are not designed to really examine the complex mechanisms and interactions among multiple independent variables (e.g., biological, behavioral, social, economic), which play out over time to affect health. Gaining a more complete understanding of these complex systems requires a new approach. Thus, there is a need for more systems science approaches (e.g., systems epidemiology), which can help better untangle the complexity in systems [1,2]. As part of the PLOS Collection “Cancer Systems Epidemiology Insights and Future Opportunities,” which covers many of the topics discussed in the National Cancer Institute (NCI) Workshop to Facilitate Cancer Systems Epidemiology Research and exemplifies the opportunities and uses of systems epidemiology approaches in cancer research [3], we describe systems science approaches and how they can be utilized in cancer research, present common misconceptions that must be overcome for systems epidemiology to realize its potential for cancer epidemiology, and describe how greater use of systems epidemiology can transform cancer research.

### Traditional cancer epidemiology top-down approaches have helped identify important associations

Epidemiology has been defined as “the study of the distribution and determinants of health-related states or events in specified populations, and the application of this study to the control of health problems [4].” Traditional epidemiological methods tend to be more top-down approaches, in which predefined designs and analytical approaches are applied to datasets. This paradigm often starts with sets of data on a disease in a specific population and tries to determine associations between risk factors and diseases and then draws inferences from the associations. This approach includes descriptive statistics of the datasets and domain-limited statistical methods to identify potential associations and trends such as linear regression, logistic regression, and survival analysis. Such methods are useful for evaluating the extent to which a variety of exposures are associated with health outcomes of interest.

Inferential statistics have helped generate important insights about cancer prevention and treatment. For example, exploring the causal role that cigarette smoking has in increasing lung cancer risk helped to develop modern statistical approaches for inference in chronic disease epidemiology and has greatly reduced cancer burden [5]. While such correlations and associations certainly do not prove cause-and-effect, they can suggest that a factor may be involved in the causal pathway of cancer, whether it is a direct cause or a sign that something else is happening. Despite their value, traditional approaches have limitations and alone cannot bridge the gaps that continue to exist in cancer research and knowledge.

### Systems epidemiology bottom-up approaches can help better understand complex mechanisms

While traditional methods help show possible associations, systems epidemiology methods are more bottom-up, aiming to rebuild the systems of interest and untangle the actual mechanisms and causal pathways involved. Such pathways may be complex, nonlinear, and dynamic, potentially spanning multiple levels, scales, and sectors. Systems epidemiology methods attempt to represent complex systems in somewhat simplified forms, distilling them to their essential elements and processes, stripping away the noise and making the system easier to understand. They can also allow for virtual testing of different circumstances, interventions, and policies that may not be possible or practical in the real-life system.

One common set of systems approaches are systems maps/diagrams that visually represent components of the system and their relationships with each other. These can show how different people’s conceptualizations or mental models of the system may be similar versus different and then identify a more comprehensive representation of the system. They can also serve as blueprints to develop subsequent systems models.

A systems map becomes a systems model when one adds quantitative representations (e.g., mathematical equations) of the relationships and processes that link the different components in the system. Once these equations are established, data are used to populate, calibrate, and validate the model. Thus, the model begins with the understanding/conceptualization of the system and not necessarily with a particular dataset. These equations can represent a situation at a particular point in time or simulate what happens over time, making it a dynamic simulation model. The equations can use specific values for a deterministic model or incorporate variability and uncertainty, making it stochastic.

Since systems models aim to recreate the system, they are quite different from traditional statistical models that try to identify associations and trends and potentially extrapolate them (Ip and colleagues describe additional differences [6]). The latter starts with the data and then identifies patterns or trends in the data according to statistical properties subject to pre-identified assumptions. Systems models are also different from other computer-driven approaches that start first with the data, such as machine learning categorization and automated feature selection, and then try to find associations and trends in the data.

Population of a systems model consists of establishing values for each parameter in each equation. Once the model is populated, calibration entails adjusting the values so that the model fits the right constraints and assumptions. Model validation determines how well the model represents what it is supposed to represent [7]. This includes face validity (experts evaluate model structure, data sources, assumptions, and results), criterion validity (how well the model can recreate real-world datasets), and convergence/divergence validity (how similar are model results to other ways of calculating such results when they should be similar and how different are they when they should differ) [7]. A key aspect of systems modeling is performing sensitivity analyses, which explore the effects of varying key model parameters. Sensitivity analyses can help reveal the major drivers or key relationships that explain observed outcomes.

A systems model can serve as a virtual laboratory to test different possibilities. Such virtual experimentation has advantages over real-world experimentation. Real-world experimentation can take considerably more time, effort, and resources. It may not even be ethical or feasible. For example, running simulations can allow you to go back in time to see what could have happened or go forward in time to see what may happen. Conducting virtual experimentation first can guide the design of real-world experiments so that these are done much more effectively and efficiently. Dynamic models can also be reverse-engineered to estimate resource requirements and their amount and type of intervention(s) necessary to achieve a desired outcome in a specified timeframe [8], and can be used to evaluate the utility of existing interventions compared to a counterfactual in which they were not implemented. By representing the actual processes in a system, simulation experiments can reveal potential unintended consequences of a policy or intervention.

**Fig 1:**
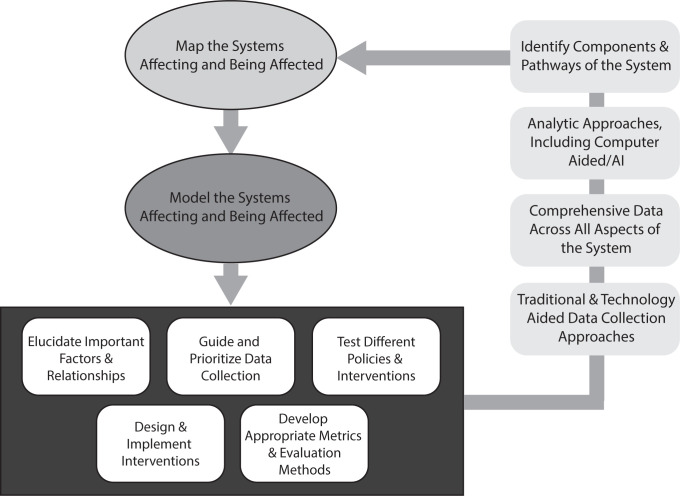
Systems approaches should be iterative.

Ideally, systems mapping and modeling should proceed in an iterative manner as illustrated by Fig 1. One does not need to come up with a perfect representation of the system at the beginning. Instead, the initial systems map and model can be a rough approximation that in turn can identify the knowledge and data gaps to then guide study designs and data collection. Once such studies and data collection yield more insights and data, the systems map and model can be updated accordingly, leading to more cycles of further refining both the systems map and model as well as the studies, data collection, and insights. This iterative process can help move toward better understanding of the system.

### Growing use of systems epidemiology methods in health research

**Fig 2:**
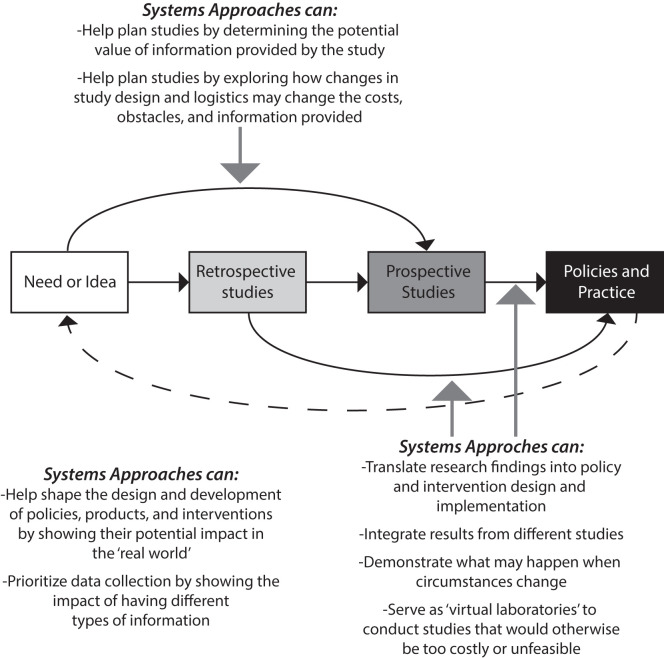
Systems modeling and approaches can and do occur at different points along the research path from idea inception to policy implementation.

There are already examples of how researchers and decision makers (e.g., stakeholders, regulators, program managers, organizational leaders, government leaders) have used such systems methods at different stages for research and decision-making, from conceptualization to development to the real-world (as Fig 2 illustrates) [9–16]. For example, computational models have helped demonstrate relationships and effects that traditional methods may have missed [17], such as how smoking cessation treatment policies resulted in the largest reductions in smoking prevalence, followed by cigarette tax increases, smoke-free air laws, and educational policies but that implementing these all in combination yielded a significantly larger reduction than any single one alone [10]. As another example, models have helped decision makers better understand the impact and cost of physical inactivity rates among youth and revealed how the type and intensity of physical activity could significantly affect the results clinical outcomes and costs [13]. Computational models have also helped show how distributing vaccines to lower income neighborhoods first during a pandemic could be more beneficial to society [15] and how cooperation among healthcare facilities in a region can lead to better overall control of an antibiotic-resistant pathogen [16].

### Systems epidemiology applications in cancer research

There are examples of systems methods assisting with cancer-related research. Initiatives such as the NCI’s Cancer Systems Biology Consortium [18] and Integrative Cancer Biology Program [19] have generated new insights. For example, systems approaches have made substantial progress in characterizing the genetics of cancer and contributions of individual intracellular pathways involved in tumor initiation and progression [20]. They have also provided a better understanding the drivers of tumor growth and cancer development and progression [21–23] as well as identifying possible cancer treatments for novel combination therapies [24].

In the field of cancer epidemiology, systems approaches have been used to inform a variety of policy making such as helping guide recommendations for cancer screening. For example, collaborative systems modeling has been used to inform the US Preventative Services Task Force’s (USPSTF) breast cancer screening recommendations [25]. Within the Cancer Intervention and Surveillance modeling Network (CISNET), 6 independently developed models evaluated mammography screening strategies in the U.S. population and helped inform decisions being made about various screening strategies [26]. Data used in CISNET models included age-specific breast cancer incidence, digital mammography performance characteristics, ER/HER2-specific treatment effects, and average and comorbidity-specific non-breast cancer causes of death, among others. Outputs of these models include reduction in mortality, breast cancer deaths averted, life-years and quality-adjusted life years (QALYs) gained and the number of screenings, false-positive screens, benign biopsies, and overdiagnosed cases (that is, cases that would not have been clinically detected in the absence of screening due to lack of progression or death). The results of these systems models showed that biennial screening strategies are the most efficient and that digital mammography screening of average-risk women aged 40 to 50 years modestly lowers mortality and extends the length of life [26]. These CISNET systems models have continued to inform policy making and to investigate emerging issues in breast cancer control including legislation about the risks of undergoing mammography on long-term breast cancer outcomes, impact of comorbidities on when screening should stop and on overdiagnosis, and the costs and benefits of transitioning to digital screening [25]. Further, CISNET models of other common cancers have shed light on the relevance of exposures such as smoking intervention in reducing lung cancer burden [27] and colorectal screening for reducing colon cancer development [28].

Additionally, the amount of whole-genome tumor sequence and biological annotation datasets have been rapidly increasing in size, number, and content. With this growth, there is a need for a systems epidemiology approach to integrate functionality across databases, methods, and analyses. An example is the development and application of software that uses a systems approach to manage, annotate, and analyze cancer mutations (using tumor data across dozens of studies and tissue types) [29]. This approach uses information from multiple different annotation sources to differentiate tumor mutations that are drivers from passengers; the drivers are then retained in a novel panel for sequencing in cell-free DNA [30]. By incorporating multiple levels of information (whole-genome sequence data), this approach outperformed conventional sequencing panel methods (e.g., based on frequency of observed mutations) in an application to prostate cancer [30,31].

### Barriers to greater use of systems epidemiology in cancer research

However, existing efforts have only scratched the surface of what systems epidemiology can do for cancer prevention and treatment. Use of systems epidemiology approaches has been limited by common misconceptions, such as those listed in Table 1, lack of training, lack of funding, lack of awareness, and institutional and professional inertia. Few universities offer systems epidemiology training programs. Systems epidemiology requires crossing over many traditional disciplines that often are siloed off from each other such as those of programmers, modelers, epidemiologists, clinicians, and policy makers. Many funding mechanisms and scientific review processes still focus on more established traditional approaches [32]. Change in general can take time. Of course, the extent to which systems epidemiology can be used depends on how well the different mechanisms involved in cancer biology and epidemiology are elucidated, how well the maps and models can represent these mechanisms, and how much the scientific community accepts such representations. These are far from unsurmountable challenges and, in fact, can grow more and more achievable with time.

**Table 1:** Common misconceptions about systems maps and models.

Misconception	Reality
A model is only as good as its data (“garbage in, garbage out”).	Systems models are much more than just the data and more about the components of the system, the mechanisms connecting them, and how all of these fit together. In fact, perfect data will rarely, if ever, be available. Therefore, systems models can help organize and contextualize existing data and guide and prioritize data collection.
Systems mapping and modeling cannot commence until the system is fully understood and the input data are fully available [letting perfect be the enemy of the good (or useful) when it comes to building the model].	Even an initial, imperfect systems map and model can provide important insights and help guide data collection and the design of subsequent studies. In fact, systems mapping and modeling should proceed in an iterative manner where any studies and data collection that result from 1 version of the systems map and model can generate more results and insight to further refine the systems map and model (see Fig 1).
The primary purpose of models is to serve as crystal balls to predict the future.	Forecasting is just one possible use of systems models. There are many other potential uses such as better understanding how components of a system interrelate and identifying key drivers of outcomes, characterizing the nature and impact of an issue [34], characterizing the potential value of different policies and interventions under varying conditions [35], and guiding data collection as well as plan and design studies (Fig 2). By simulating how the system will react to interventions under various scenarios, systems models can serve as planning tools to identify solutions that are more robust to uncertainty and variable conditions.
A single model is sufficient to address a problem.	A single observational study or clinical trial is not enough to address a problem. Similarly, multiple models, each with different structures, inputs, perspectives, assumptions, and strengths and limitations are needed. One can use comparative modeling, that is, developing multiple systems models, to address the same problem and comparing their approaches and results. Where model results converge, confidence that the results are robust to different assumptions increases and where models diverge in their results, each model’s assumptions can be systematically examined to understand what differences are responsible, yielding new insights.
One model can solve any problem (the hammer looking for the nail problem).	There are many different types of systems models and methods (e.g., decision analytic, compartment, system dynamics, network, and agent-based), each with its strengths and limitations. Even models within the same method can be very different. Thus, one should not start with a modeling method and try to force the representations of the issue in the model. Rather, the issue/question and the systems involved should determine the type of systems modeling method(s)/model(s) used.
All models are the same, and systems models are not different from other types of models.	The term model encompasses a wide range of possibilities; thus, it is not enough to say a “model” was used to generate results or a solution. Systems models are more about the components of the system, the mechanisms connecting them, and how all of these fit together. They use a bottom-up approach and aim to rebuild a system of interest and untangle the actual mechanisms and causal pathways involved. Even different types of systems maps and models are different, and the value of each to address a specific issue/question depends on the map/model’s purpose, what kind of systems map or model was used and the approach used to build it, its data and structure, its strengths and limitations.
Believing the George Box quote that “all models are wrong, some are useful” means that models are less real than other research methods.	Every scientific study to some degree is a simplification of reality; no study whether a systems modeling study, clinical trial, or cohort study can truly represent all the diversity and complexity of real-life. Therefore, using the criteria of Box, all types of studies are “wrong.”
A single model output can provide enough information.	Just as a single measure cannot tell you the health of a person, a single output without context cannot tell you much about a system. Instead to adequately represent a system, multiple different types of outputs are needed.
A statistical model is the same thing as a systems model.	Statistical models are more top-down approaches that start with the data and try to identify associations and trends but cannot determine cause and effect; whereas systems models are more bottom-up approaches that actually try to represent and rebuild the system of interest including its causal pathways and mechanisms.
It is enough to simply throw some engineers, computer scientists, or modelers at the problem.	Just because someone is a computer scientist or an engineer does not necessarily mean they understand and appreciate systems modeling. Systems mapping and modeling is its own discipline that crosses many different disciplines (e.g., computing, health, public health, epidemiology, medicine). It requires not only being able to develop and write code, but also the conceptualization and understanding of the system and the translation of it into a proper structure and set of equations.
Systems are too complex to represent.	Systems maps and models do not need to include every possible detail of a system. Instead, the goal is to identify key components and causal pathways.
Developing a systems model does not require substantial time, effort, and resources.	While conducting clinical, observational, or laboratory studies may require more time, effort, and resources, the quality and utility of a systems model does heavily depend on the time, effort, and resources spent. People unfamiliar with systems modeling may substantially underestimate what is involved.
The value of a model is only in the answers that it provides.	Many times, the value of a systems map or model is in the questions that it raises. Systems maps and models can help identify what data and knowledge is missing and its relative value in reducing uncertainty in model outputs. Additionally, systems models can help identify thresholds or key inflection points at which things may occur or for which the value of a policy or intervention changes.
The perspective of the model is not important.	A systems map or model developed for the perspective of a particular decision maker (e.g., individual patient, a health care professional, third-party payer, society) does not necessarily apply to other decision makers. In fact, the results and potential solutions can differ significantly by perspective.

### Greater use of systems epidemiology can transform cancer research

Our society is at an inflection point where there is now more data from different, disparate, and wide-ranging sources and there is wide availability of analytic tools with greater computational resources and power. We are no longer limited to analyzing a single dataset or study population at a time. Systems epidemiology methods can help link different, disparate data and make better use of data that may have been viewed as imperfect in the past. While research in the past several decades has resulted in more effective prevention and treatment measures, there are a number of areas where progress has stalled. For example, some cancers (e.g., skin, liver) have been on the rise and others (e.g., pancreatic, liver, esophageal) continue to have poor cure rates [33]. This suggests that the causal pathways may be more complex than realized and that key factors and processes are not being addressed. With a greater understanding of the capabilities of systems epidemiology and a greater investment of resources, it is our hope that systems epidemiology may be able to better elucidate these causal pathways and lead to more effective prevention and treatment measures. Moreover, significant disparities exist in many cancer diagnoses and outcomes and risk, and the course of cancer can vary substantially among different people and populations. Therefore, one-size-fits-all approaches that are driven by standard designs and analytical approaches may not work adequately. Systems epidemiology can help identify and develop more tailored approaches and move toward precision medicine for cancer prevention and treatment. In addition, even when cancer treatments are effective, they can have risks and side effects. Systems epidemiology can help elucidate what may be leading to these risks and side effects and help develop better treatments. Finally, with a greater recognition of the many existing constraints, systems epidemiology can help decision makers such as clinicians, public health officials, policy makers, and third-party payers prioritize initiatives, save time, effort, and money, and better allocate limited resources among different cancer research, prevention, and treatment options.

## Summary

Systems epidemiology methods, such as mapping and dynamic simulation modeling, are designed to gain understanding of complex phenomena through simplified representation and virtual experimentation. These methods have proven to be valuable complements to other methods of inquiry in other health domains but have been underutilized to date in cancer epidemiology. With increasing availability of high-performance computers and sophisticated analytical tools, systems epidemiology has enormous potential to expand research on cancer prevention, treatment, and control by helping untangle the complexities in cancer epidemiology. The benefits of using systems epidemiology include a better understanding of a problem’s impact over time, identification of leverage points for intervening in the system, and trade-offs and consequences of policy decisions. When adopting systems epidemiology methods, researchers should be aware of the common misconceptions that need to be overcome. Systems epidemiology can transform cancer research by helping identify and develop more tailored approaches to move toward precision medicine for cancer prevention and treatment.

## Abbreviations

CISNET: Cancer Intervention and Surveillance modeling Network
NCI: National Cancer Institute
QALY: quality-adjusted life year
USPSTF: US Preventative Services Task Force
